# Supplementary material for: A short-lived peptide signal regulates cell-to-cell communication in Listeria monocytogenes
Source: Commun Biol. 2024 Aug 3;7:942. doi: 10.1038/s42003-024-06623-6 (PMC11297923; doi:10.1038/s42003-024-06623-6)
Supplement: Supplementary file 3 — Description of Additional Supplementary Files [file 42003_2024_6623_MOESM3_ESM.pdf]

Description of Additional Supplementary Files

File name: Supplementary Data 1
Description: Data points used for graphs depicting rearrangement rate of P2 to P3 and hypothetical P2 decay.

File name: Supplementary Data 2
Description: All measurements of luminescence and optical density at 600 nm used for graphs depicting luciferase reporter strain assay results.

File name: Supplementary Data 3
Description: Relevant extracted ion chromatograms and mass spectra used to assess stability of *N,N*-dimethyl P2 (1) after 22 hrs at pH 7 and 37 degrees Celcius.

File name: Supplementary Data 4
Description: Relevant extracted ion chromatograms and mass spectra used to assess stability of P2 C1Ser (8) after 22 hrs at pH 7 and 37 degrees Celcius.

File name: Supplementary Data 5
Description: All raw Cq values from qPCR runs and data treatment.
